# Cross-Cultural Adaptation and Validation of the Malay Version of Sports Motivation Scale-II

**DOI:** 10.3390/ijerph182111694

**Published:** 2021-11-07

**Authors:** Ngien-Siong Chin, Guo Chen Liew, Yee Cheng Kueh, Hairul Anuar Hashim, Vincent Tee, Garry Kuan

**Affiliations:** 1 Physical Education and Health Department, Institute of Teacher Education Batu Lintang Campus, Kuching 93200, Sarawak, Malaysia; ngiensiong@gmail.com; 2 Exercise and Sports Science Programme, School of Health Sciences, Universiti Sains Malaysia, Kubang Kerian 16150, Kelantan, Malaysia; steven_liew2000@hotmail.com (G.C.L.); hairulkb@usm.my (H.A.H.); 3 Sarawak Education Department, Kuching 93050, Sarawak, Malaysia; 4 Biostatistics and Research Methodology Unit, School of Medical Sciences, Universiti Sains Malaysia, Kubang Kerian 16150, Kelantan, Malaysia; vincenttee1996@gmail.com; 5 Department of Life Sciences, Brunel University London, London UB8 3PH, UK

**Keywords:** sports, motivation, athletes, Malay, Malaysian

## Abstract

The 18-item Sport Motivation Scale (SMS-II) is one of the most-utilised scales measuring athletes’ motivation and its psychometric properties. However, we found no Malay version used to examine the Malaysian multi-ethnic population. Thus, the study aimed to translate and validate the SMS-II into the Malay version using confirmatory factor analysis. A total of 436 (16.44 ± 1.22) state athletes were asked to complete the SMS-II Malay version, which utilised the forward–backwards translation method. The results showed an acceptable fit with the data (CFI = 0.906, SRMR = 0.064; RMSEA = 0.056) and internal consistency, with a Cronbach’s alpha value that exceeded 0.50, which supported its usage for the assessment of motivation among the multi-ethnic Malaysian athletes. The Cronbach’s alpha values of all the factors were satisfactory, except for introjected regulation. Thus, further studies are needed to improve the reliability of such factors. Nonetheless, the Malay version of SMS-II was found to be valid and reliable for assessing the level of motivation of the multi-ethnic Malaysian athletes.

## 1. Introduction

Motivation is often described as the drive that pushes the intensity of one’s training and commitment in sports and physical activity [1]. This is important for individuals who are concerned about their health and wellbeing, or improving their performance in a competition [1]. Motivation has been the subject of investigation as researchers are curious about the internal factors driving individuals to “do more” and “practice harder” [2]. Motivation, however, is difficult to measure as it is a subjective and latent variable. Early studies have tried to assess it through verbal reports [3] and by observing the time that a study participant spent on a specific task [4]. Researchers, however, have tried to develop effective measurement tools to better quantify and determine a person’s motivation in the fields of sports and exercise. Clearly, a more robust method with practical theoretical frameworks is needed to understand the nature of one’s motivation and behaviour.

Numerous frameworks have been used to illustrate the influence of motivation in sports and physical activities. Social-cognitive theories have often been used in studies about youth, sports, behaviour, and motivation in recent decades [5]. For instance, the achievement goal theory proposed by Nicholls [6], which focused on the task- and ego-specific goals concerning a person’s motivation in order is a common motivational theory. This theory states that individuals who are task-specific are likely to hone their skills to achieve better results in sports, whereas individuals who are ego-specific tend to get motivated to perform better only to avoid criticism. The transtheoretical model is an integrative model that focuses on intentional behavioral change through a balanced and staged process [7,8,9], but it does not specify motivation per se. The self-determination theory (SDT) of Deci and Ryan [10] focuses on the intrinsic and extrinsic motivation driving a person’s behavior. We believe that SDT [10], which provides a robust and comprehensive framework in multifaceted dimensions in sports, physical activities, physical education, and exercise [11,12], is more applicable to a country with a multi-ethnic and multi-cultural population, such as Malaysia.

SDT explains that motivation is a “construct that can be ordered on a continuum”. The continuum begins from “amotivation”, where an individual is non-motivational or does not have any intention, and progresses to “extrinsic motivation”, where motivation originating from an external source surfaces, and then to “intrinsic motivation”, where self-determined motivation stemming from inside the person emerges [13]. The theory helps to narrate how each autonomous form of motivation (self-determined) influences our physical construct [12] and sports activities [14].

### 1.1. Sports Motivation Scale

The Sports Motivation Scale (SMS) played a major role in measuring an athlete’s motivation within the research community before the revised version emerged. In a bibliometric analysis [3], six highly cited questionnaires on motivation were identified. Among these were the Intrinsic Motivation Inventory [15], which focuses only on intrinsic motivation; the Situational Motivational Scale [16], which focuses on the unidimensionality of intrinsic motivation and the reasons for individuals’ involvement in a specific activity; and the 36-item Behavioral Regulation in Sport Questionnaire (BRSQ) [17], which was specifically designed for use by competitive athletes.

SMS had 28 items on seven factors measuring three types of intrinsic motivation (IM; IM to know, IM to experience stimulation, and IM to accomplish), three types of extrinsic regulation (external regulation, introjected regulation, and identified regulation), and amotivated regulation. The factor structures and scale reliability were pooled from a group of French-speaking Canadian university student athletes participating in several team-based and individual sports [2]. To ascertain the scale’s psychometric properties, both versions underwent a comprehensive and extensive validation process through structural equation modeling analysis [18].

Despite the extensive use of SMS in the field of sports psychology [19], its drawbacks did not go unnoticed. It had poor correlations for the seven-factor model based on a sample of American college athletes. Some studies had highlighted its inability to validate the factor structures on non-native English speakers and athletes from different age groups. Additionally, it is worth noting that the initial version of SMS did not include the integrated-regulation measure. Furthermore, some researchers who used it encountered problems with the psychometric properties of the IM subscales regardless of their relatively high correlations [20,21]. This diminished SMS’s ability to include the theoretical construct in SDT.

### 1.2. Sport Motivation Scale-II (SMS-II)

Consequently, a revised SMS (SMS-II) [3] was created to address the shortcomings of the original version [19]. The revised version included several modifications. First, the authors reduced the number of dimensions by considering intrinsic motivation as a singular dimension encompassing the three original types of intrinsic motivation. Second, the original 28 items were reduced, resulting in a version with three items per factor. Third, the integrated-regulation construct, which is different from the intrinsic-motivation and identified-regulation constructs, was added. Lastly, some of the items were altered or removed to facilitate the conceptual delivery.

SMS-II consists of six factors: intrinsic motivation, integrated regulation, identified regulation, introjected regulation, external regulation, and amotivation. In short, it is a more comprehensive instrument that includes all the constructs in the new SDT framework. It uses the introductory sentence: “Why do you practice your sport?”, and a 7-point Likert-type response scale ranging from 1 (“does not correspond at all”) to 7 (“corresponds completely”). SMS-II has a good factor structure (Root Mean Square Error of Approximation = 0.05; Comparative Fit Index = 0.93; Tucker-Lewis Index = 0.91) according to the confirmatory factor analysis (CFA) reported by Pelletier et al. [16]’s Study 2, and good Cronbach’s alpha coefficients (0.70–0.88) in Study 1. The results of the validation of the revised scale indicate that SMS-II performs well and better than the original scale.

Unlike the Motives for Physical Activity Measure-Revised (MPAM-R) scale composed by Ryan et al. [22], SMS-II has been translated and validated into several languages, demonstrating good divergent validity and internal consistency among youth athletes. It has been translated into and validated in Chinese [23], Spanish [24], Portuguese [25], and French [26] via CFA. To the best of our knowledge, the 30-item MPAM-R scale, which requires a longer application time, has been translated and cross-culturally adapted in only four studies and two languages. Moreover, the results obtained by Gonclaves and Alchieri [27] revealed that the items were not acceptable on the basis of the CFA criteria whereas the study by Albuquerque et al. [28] used a weighted factor loading method less common than CFA. The present study translated SMS-II into Malay and examined the validity and reliability of the Malay version using construct validity, internal consistency, and test-retest reliability to ensure that it can be used to measure the types of motivation in the Malaysian population.

## 2. Materials and Methods

### 2.1. Participants

The participants comprised 436 athletes between 14 to 21 years old (16.44 ± 1.22), who actively represented a variety of sports. Participants consisted of both males (*n* = 299; 68.6%) and females (*n* = 137; 31.4%). The athletes were from various sports that had competed at the state, national and international levels. All participants had adequate comprehensive and speaking skills (passed an elementary level examination) in the Malay language. Participants were informed of the objectives and their ethical rights during the consenting procedure. The study was voluntary and the name of the participants was kept anonymous throughout the study. Participants were free to terminate their participation at any point of the study without any penalty.

### 2.2. Measures

#### 2.2.1. Sports Assessment

The questions were used to obtain information on their gender, ethnicity, and age. Furthermore, the questionnaires were aimed at quantifying what type of sports the participants were involved in; the duration of their training; their level of involvement (e.g., district, state, national, and international); their socio-demographic information.

#### 2.2.2. The Sports Motivation Scale-II (SMS-II)

The SMS-II is an 18-item scale that comprises 6 subscales, i.e., intrinsic motivation, integrated motivation, identified regulation, introjected regulation, external regulation, and amotivation with three items attached in each subscale. All the items were rated on a 7-point Likert scale from 1 point (not true at all) to 7 points (very true).

### 2.3. Questionnaire Translation

The original version of the SMS-II [19] was translated according to the translation guidelines [29]. First, the SMS-II was translated from English to Bahasa Melayu by two independent bilingual translators who were well-versed in English and Bahasa Melayu. Then, the translated questionnaire was back-translated by two independent bilingual translators to the English version. The backward translation and the original questionnaire were compared, reviewed, and verified by five bilingual experts from the fields of sport psychology, sport science, and physical education. The experts then finalised the scale in terms of item clarity which reflected cultural objectivity, comprehensibility, and conceptual equivalence. The experts also matched with its corresponding items in the original English version, and conducted further assessments to ascertain whether they were culturally appropriate for the Malaysian population. Finally, a sample of 15 young state athletes was invited to access the items’ clarity, understanding, and comprehension. The young athletes’ feedback was similar to one another and required no revisions.

### 2.4. Data Collection

The study received ethical approval from the Human Research Ethics Committee of the Universiti Sains Malaysia (USM/JEPeM/16020085) and was conducted following the guidelines of the International Declaration of Helsinki. The study was a cross-sectional design and implemented between April and December 2019. The participants were invited to participate in the study, agreed, and provided their informed consent form. They were assured that their participation in the study was confidential.

### 2.5. Statistical Analysis

The Confirmatory Factor Analysis (CFA) was performed to verify the six-factor structures of the Malay version of the SMS-II in terms of construct validity utilising statistical software Mplus 8 (Muthén & Muthén, Los Angeles, CA, USA). Data normality was tested using skewness and kurtosis. In this study, the MLM estimator was selected for CFA as it could withstand the non-normality distribution of data and provided estimates with standard errors that included a mean adjusted chi-square statistic [30]. Internal consistency coefficients of the 6-factor model were evaluated using Cronbach’s α, with scores between 0.45 to 0.98 and were described as acceptable, although a value more than 0.60 is generally recommended [31]. Significant factor loading of 0.50 and above with a modification index was used as a criterion to retain or delete items from the measurement model [32]. Based on the 18 items as a 6-factor structure measurement model in the present study, the following fit indices and fit values were used: the comparative fit index (CFI) and Tucker and Lewis index (TLI), with a recommended value higher than 0.90, the root mean square error of approximation (RMSEA), with the recommended value of less than 0.07 and the standardised root mean square (SRMR), with the recommended value of less than 0.08 [33,34]. The test–retest reliability analysis was analysed using the Intraclass Correlation Coefficient (ICC) on 40 athletes over a week apart. Next, the socio-demographic data were self-reported. Lastly, descriptive statistics were utilised to describe the characteristics of the participants.

## 3. Results

### 3.1. Socio-Demographic Characteristics

A total of 436 athletes participated in this study. The mean age of the sample was 16.44 (*SD* = 1.22), with ages that ranged from 14 to 21 years old. The majority of the participants were Malay athletes (*n* = 301; 69.0%), followed by aboriginal Sabah/Sarawak athletes (*n* = 78; 17.9%), Chinese athletes (*n* = 51; 11.7%), other ethnic athletes (*n* = 4; 0.9%) and Indian athletes (*n* = 2; 0.5%).

All of the athletes were from different types of sports and competed at different levels (e.g., competitive or recreational) in Malaysia. From the sample, 23 types of sports athletes were involved and 228 (53.5%) of the participants were involved in team-based sports, while 198 (46.5%) were involved in individual sports. The demographic characteristics of the athletes are shown in Table 1.

### 3.2. Validity and Reliability

**Figure 1:**
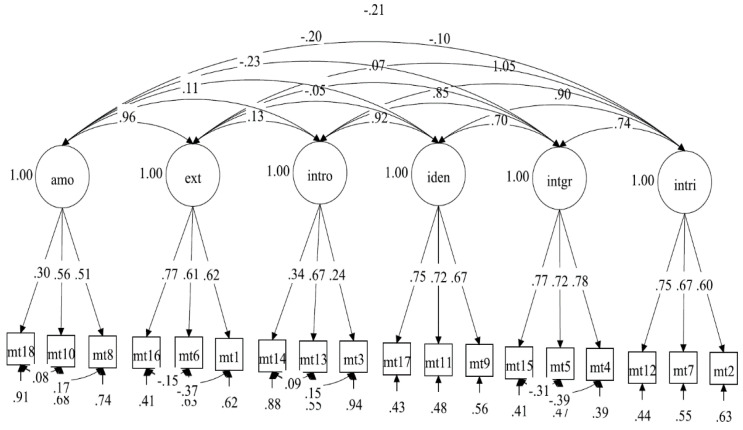
The standardised item loading for the measurement model. Note. amo = amotivated, ext = external, intro = introjected, iden = identified, intgr = integrated, intri = intrinsic.

The hypothesized measurement model of the SMS-II Malay version comprised 18 items with 6 factors: amotivation (3 items), external regulation (3 items), introjected regulation (3 items), identified (3 items), integrated regulation (3 items), and intrinsic motivation (3 items). The results of the initial hypothesised model of the SMS-II Malay version displayed an acceptable fit with the data (RMSEA = 0.056, CFI = 0.906, TLI = 0.880, SRMR = 0.064). Subsequent model modifications were made by adding a correlation among the items’ residuals within the same factor, which resulted in good fit indices (RMSEA = 0.053, CFI = 0.923, TLI = 0.895, SRMR = 0.062). Table 2 displayed a summary of model fit indices in the SMS-II scale. Figure 1 shows the standardised item loading of the measurement model. Additionally, the reliability indexes displayed adequate internal consistency for all six factors.

The internal consistency coefficients measured by Cronbach’s alpha were: 0.718 for intrinsic, 0.734 for integrated, 0.752 for identified, 0.462 for introjected, 0.617 for external, and 0.526 for amotivation (refer to Table 3). These values revealed that the SMS had acceptable to excellent stability over time. The ICC values for the six subscales were 0.90 (Intrinsic), 0.83 (Integrated), 0.53 (Identified), 0.80 (Introjected), 0.84 (External), and 0.90 (Amotivaiton) based on values between 0.4 and 0.7, between 0.70 and 0.9, and greater than 0.90 indicating moderate, good and strong reliability, respectively [35,36].

## 4. Discussion

The present study aimed to translate SMS-II into Malay and validate the psychometric properties of the Malay version among Malaysian athletes. The Malay language, with more than 200 million speakers, is ranked the world’s fifth most widely spoken language [37]. The language is still growing, and its share of the languages spoken by the world’s population is expected to increase. Notwithstanding the widespread applicability of SDT in deciphering athletes’ motivation, there were no valid tools available in the Malaysian language for evaluating the theoretical frameworks proposed in SMS-II. The closest assessment tool available with regard to the Malaysian context was the Malay version of the Recreational Exercise Motivation Measure by Kueh et al. [38]. It has shortcomings, however, in its relationship with the SDT framework, and mainly focuses on undergraduate students and their motivation for recreational exercise.

For exploring the psychometric properties of the Malay version of SMS-II, we used the construct validity in CFA, which measures the extent to which the set of items reflects the theoretical construct that the items are supposed to measure [33]. A similar validation process was applied for other translated versions of SMS-II [2,23,24,26], and satisfactory results were obtained. The present study aimed to confirm rather than explore the structure of the Malay version of SMS-II. Thus, CFA was used instead of exploratory factor analysis. Some items’ residuals were allowed to be correlated in the final CFA model, but this was carried out within the same factor, and the theoretical meanings were determined. Additionally, the contents of the items may be related or similar within the same factor. The items’ residual covariances indicate that the paired items have some common variances that are not specified in the model [34]. Therefore, after the CFA model was respecified by adding the covariances of the items’ residuals within the same factor, the model fitness improved, and the final model fit the data well. SMS-II was thus found to fit the data well, and the final model retained all the 18 items, with strong factor loadings (above 0.30) on their respective factors. Furthermore, the results provided substantial evidence for measurement and structural invariance.

In addition, the Cronbach’s alpha values, which ranged from 0.462 to 0.752, revealed acceptable reliability for all the factors. Introjected regulation, however, with a Cronbach’s alpha value of 0.46, close to the recommended value of 0.50, showed only moderate reliability [39]. The Cronbach’s alpha value of this factor in the current study may be lower than that in the study by Pelletier et al. [19,23]; however, it is almost similar to those in the studies by Ntoumanis and colleagues [40,41,42,43,44]. The item also showed good stability over two time periods, with an ICC value of 0.80. Considering that young athletes are in the period of puberty, they may be concerned about their outlook, a form of introjected regulation [38,45,46]. We thus decided not to exclude this item in the study.

Studies have shown that introjected regulation or motivation was relatively lower among younger athletes involved in team-based sports [47,48]. Furthermore, the sample of interest in such studies consisted of novice athletes with professional coaching and training schedules that had sufficient breaks and break intervals. Gillison et al. [49] demonstrated that the introjected regulation in boys was linked to social factors (avoiding social disapproval and attaining ego enhancement), whereas in girls it focused on partial internalization (appearance and health issues). In our sample population, 68.6% were males, and we believe that this somehow influenced the results of our study. Further supporting the notion that the above result may have stemmed from a sample-specific issue is the possibility that cultural differences among the Asian and Western populations may play a role in this matter. For instance, in one of the items (in English, “Because I would not feel worthwhile if I did not do it”), the word *kepuasan* in Bahasa Melayu may imply satisfaction (gaining pleasure or contentment from the activity), which may point to an intrinsic motivation. The word *kepuasan* may also imply being worthwhile, as the action justifies the investment of time or interest in Malaysian culture. Future studies can implore the simplex-like difference and examine the linguistic, cultural, and religious invariances among the Asian and Western countries. Also, Standage et al. [47] reiterated that the context of introjected regulation in physical activity may differ from the antecedents of the general context. This is partly due to the abundance of social advocates and health institutions promoting the benefits of exercising. Concurrently, this may also warrant increasing or reconstructing the number of items in future studies to achieve a higher Cronbach’s alpha value of the factor.

The present study had several limitations. It was conducted among younger participants (mean age = 16.44) and athletes with diverse levels of sports involvement. Hence, the questionnaire may not be applicable to participants from an older age group or to Malaysians with a specific level of sports involvement. It is recommended that future studies be conducted among participants from heterogenous age groups and with different levels of sports involvement. We also acknowledge the possibility that there was a response bias due to the nature of the self-report survey. As such, the participants in future studies need to be constantly reminded that they should respond with honesty and that their responses will be kept confidential. The future studies should also include more items in the two subscales and should conduct a retest for their reliability. Additionally, future studies can hold an interview session and analyze the data obtained by using interpretative phenomenological analysis to further explore the participants’ experience in relation to introjected regulation toward sports. Lastly, it would be interesting to conduct a concurrent validation to assess and compare the psychometric properties of the Malaysian population using different scales and instruments stemming from the SDT framework. Lonsdale et al. [50] mentioned that “scale development” is an “ongoing process.” Therefore, more research is warranted to investigate the advancement of the scale.

## 5. Conclusions

To conclude, the results of the present study show the validity and reliability of the Malay version of SMS-II. We recommend its use in measuring the motivation levels of young Malaysian athletes. We also propose the commencement of a longitudinal study to examine the stability of the cross-time relations for the scale and the changes over time in the administration of a comprehensive intervention focusing on athletes’ discrete motivation. Additionally, it would be interesting for future studies to explore the difference in the motivation construct among athletes from different cultural and ethnic groups (Malay, Chinese, Indians, and aboriginals) in Malaysia. Furthermore, we would recommend the exploration of the comparative psychometric properties of SMS-6, BRSQ, and SMS-II among Malaysian athletes in the future.

## Figures and Tables

**Table 1:** The demographic characteristics of the athletes who are representative of state teams (*n* = 436).

Characteristics	Frequency	Percentage	Mean (*SD*)
Gender			
Male	299	68.6	
Female	137	31.4	
Age (years)			16.44 (1.22)
Ethnicity			
Malay	301	69	
Bumiputera	78	17.9	
Chinese	51	11.7	
Indian	2	0.5	
Others	4	0	
Types of sports			
Badminton	10	2.3	
Cycling	15	3.4	
Bodybuilding	1	0.2	
Handball	4	0.9	
Netball	23	5.3	
Basketball	14	3.2	
Football	130	29.8	
Bowling	9	2.1	
Futsal	37	8.5	
Gimrama	6	1.4	
Hoki	15	3.4	
Karate	1	0.2	
Archery	10	2.3	
Muay Thai	1	0.2	
Athletics	98	22.5	
Ping Pong	3	0.7	
Rugby	3	0.7	
Sepak Takraw	2	0.5	
Silat	8	1.8	
Squash	10	2.3	
Taekwondo	10	2.3	
Boxing	12	2.8	
Wushu	14	3.2	

**Table 2:** Fit Indices for six models in SMS-II.

Model	RMSEA (90% CI)	CFI	TLI	SRMR
Model-1 (Initial)	0.056 (0.050, 0.063)	0.906	0.880	0.064
Model-2 ^a^	0.053 (0.046, 0.059)	0.923	0.895	0.062

^a^ 1-Factor measurement model with correlated items residual; Q5 and Q15, Q4 and Q15, Q3 and Q14, Q13 and Q14, Q1 and Q16, Q6 and Q16, Q8 and Q18, Q10 and Q18.

**Table 3:** Internal consistency and test–retest reliabilities of SMS-II dimensions.

Subscales	Cronbach’s Alpha	ICC (95% CI)
Intrinsic motivation	0.718	0.90
Integrated regulation	0.734	0.83
Identified regulation	0.752	0.53
Introjected regulation	0.462	0.80
External regulation	0.617	0.84
Amotivation	0.526	0.90

Abbreviations: CI = Confidence Interval, ICC = Intraclass correlation coefficient.

## Data Availability

Data are available upon request from the authors.
